# Characteristics of sexual violence against adolescent girls and adult women

**DOI:** 10.1186/1472-6874-14-15

**Published:** 2014-01-22

**Authors:** Márcia de Toledo Blake, Jefferson Drezett, Maria Auxiliadora Vertamatti, Fernando Adami, Vitor E Valenti, Adriana Costa Paiva, Joseval Martins Viana, Daniela Pedroso, Luiz Carlos de Abreu

**Affiliations:** 1 Laboratório de Delineamento de Estudos e Escrita Científica. Departamento de Ciências Básicas, Faculdade de Medicina do ABC, Av. Príncipe de Gales, 821, 09060-650 Santo André, SP, Brazil; 2 Hospital Pérola Byington, São Paulo, SP, Brazil; 3 Departamento de Fonoaudiologia, Faculdade de Filosofia e Ciências, UNESP, Av. Hygino Muzzi Filho, 737, 17525-900 Marília, SP, Brasil; 4 Departamento de Saúde Materno-Infantil. Faculdade de Saúde Pública, Universidade de São Paulo, São Paulo, SP, Brazil; 5 Universidade Federal do Pará. Capus Soure, Ilha de Marajó, PA, Brazil

**Keywords:** Sex offence, Criminal, Adolescent

## Abstract

**Background:**

Sexual violence is considered a serious violation of human rights which affects mainly young women and adolescents. There is little information about the conditions under which sexual offences occur. We evaluated characteristics of sexual violence against adolescent girls and adult women.

**Method:**

This is a quantitative, retrospective, descriptive study of sexual violence against adolescent girls and adult women. Analyses were carried out on data collected from 1118 women, 546 adolescents (10-19 years) and 572 adults (≥ 20 years), with a complaint of rape treated at Hospital Pérola Byington, São Paulo, between 1994 and 1999. The age limit of the adolescent sample met the World Health Organization’s (WHO) criteria. We analyzed the type of sexual contact, degree of intimidation, perpetrator and activity of the victim during the approach.

**Results:**

Crimes without penetration were five times more frequent in adolescents and use of threats of death or intimidation was common in both groups. Mental illness was more prevalent in adult victims and the majority of adolescent victims were aged <14 years. Uncle and stepfather perpetrators were more frequent among adolescents and partners or former intimate partners in adult women. In most cases the approach occurred in public places, although sex crimes at the perpetrator’s residence were more frequent amongst adolescents.

**Conclusions:**

Although children and adolescents require the same intervention measures and legal protection, a considerable proportion of adolescent sex offenders can face conditions similar to those of adult women.

## Background

Violence is one of the leading causes of morbidity and mortality in young people. While homicides predominate in males, sexual violence affects women severely, causing physical and psychological sequelae that make them vulnerable to health problems [1]. These women face significant risk of genital and extragenital trauma, lethal outcome, unwanted pregnancy, sexual dysfunction and sexually transmitted diseases (STD) [2].

The Fourth International Conference on Women held in Beijing in 1995, recognized the right of women to decide freely about their fertility and sexuality, without suffering any form of coercion, discrimination or violence [3]. However, the violation of this right occurs in almost all societies and cultures, particularly those in which women are still firmly subordinated to the arbitrary decisions of men [4].

Sexual violence is an extreme restriction of the sexual and reproductive autonomy of women [5]. Although it is largely hidden by the victims, it is estimated that 12 million people around the world face sexual violence every year [6]. Young people are the most frequent victims of sexual violence, it is generally thought that 12% to 25% of girls and 8% to 10% of boys under 18 years of age will suffer sexual violence [7].

In Colombia, a national sample study found 20% of women had a history of sexual violence [8]. Of 1400 high school students in Ethiopia, 5% reported having suffered sexual violence [9]. In Malaysia, 8.3% of medical students reported suffering some form of sexual abuse during childhood [10]. In Sierra Leone, a survey of 144 women found that almost 50% had been subjected to sexual abuse and genital mutilation [11].

In a systematic review of African studies from 2000 to 2010, a high prevalence of intimate partner violence was associated with higher risk of human immunodeficiency virus (HIV) infection, low socioeconomic status, younger age of woman, and use of alcohol and illicit drugs by the victim’s partner [12].

Despite these indicators, developing countries rarely have statistics on the problem. In Brazil, the official records of public security departments indicate 15.9 cases of sexual violence per 100 thousand inhabitants, many times less than that seen in population studies. Furthermore, the prevalence of sexual crimes is subject to regional variations, with a greater impact in populations with lower levels of social and economic development [13].

Women who suffer sexual violence need attention for various medical conditions, psychological treatment, guidance on legal issues, social support, advice on prevention of unwanted pregnancy and prophylaxis against STDs [13]. In recent years, there has been an increase in the number of victims of sexual violence in urban centres who use health services early [14].

The metropolitan region of São Paulo is the fourth largest urban conglomeration in the world, with 29 million people who face situations of crime in large urban areas. However, we still lack information about the conditions in which sexual crimes occur, the characteristics of perpetrators and victims. Additionally, adolescent victims of sexual violence are usually grouped with children in the analyses, because of their common age-related vulnerability and its legal implications.

Adolescents however, may experience sexual violence by mechanisms similar to those used against adult women, especially sexual crimes committed by perpetrators unknown to the victim and crime associated with urban violence [14]. Few investigations have compared sociodemographic variables and the dynamics of sexual offences among adult women and adolescents in these circumstances. The objective of this study therefore is to compare characteristics of sexual offences against adolescent and adult women.

## Method

### Study design

This was a quantitative, retrospective, descriptive study of 1,118 female victims of sexual violence from the metropolitan area of São Paulo. Data collection took place at the Núcleo de Violência Sexual e Aborto Legal do Hospital Pérola Byington between August 1994 and December 2000. For convenience the sample was divided into two groups; 546 adolescents (aged ≥ 10 and <20 years) and 572 adult women (age ≥ 20 years).

### Criteria for selection and inclusion of subjects

The inclusion criterion was the crime of sexual abuse presented by the patient or her legal representative consistent with the provisions of Articles 213 and 217-A of Brazilian criminal law. Article 213 criminalizes as rape any non-consensual sexual act committed with the use of physical force or serious threat. Article 217-A, dealing with rape of vulnerable individuals, covers sexual acts against children under 14 years or against those of any age who cannot offer resistance or give consent [13]. Police notification or the results of a forensic examination were not preconditions for inclusion.

The concept of rape of the vulnerable was applied only in cases where there was no serious threat or use of physical force. We included in this category girls aged <14 years, mentally disabled women and women with any other condition inhibiting their ability to resist [13].

The age limit adopted for the adolescent group was different from that defined by MeSH terms (13-18 years), but in line with the criterion used by the WHO, as regulated by the Brazilian Ministry of Health to meet legal definitions of sex crimes. The study period was chosen for convenience of data collection. However, there has been no significant change in the Brazilian criminal law or in the statistics on sexual violence since our data were collected.

### Instruments and analysis of data

The data used in the study were drawn from the standardized form used by the interdisciplinary care team and were transferred to a simplified form. Data were entered into EpiInfo6, version 6.04b. The verification of the consistency of the data was performed by double entry comparison of files and discrepancies were corrected.

### Outcome variables

The outcome measures in both age groups included the frequency of occurrence different types of sexual act, form of intimidation used by the perpetrator, perpetrator characteristic and the victim’s activity at the time the approach. Sexual acts were categorized as vaginal, anal or oral penetration, practiced alone or in combination; or other non-consensual sexual contact without penetration. Under intimidation we included strategies described in Brazilian criminal law: serious threat, use of violence, and rape of the vulnerable [13].

The perpetrator of sexual violence was characterized according to whether he acted alone or in a group, whether he was known to the victim, and whether he was related to her. The activity and location of the victim at the time of the perpetrator’s approach were categorized as: commuting to work or school, daily activity in the community, at the residence of the victim or perpetrator, leisure or recreation, or workplace.

### Statistical analysis

Frequency tables were constructed for data analysis, and the Chi-squared test (χ^2^) for contingency and association was applied using p ≤ 0.05 as the criterion for rejection of the null hypothesis. The Odds Ratio was used for variables in each age group, with 95% confidence intervals (CIs).

### Ethical aspects

The study was approved by the Research Ethics Committee of the Hospital Pérola Byington, protocol n° 034/11, in September 2011 and conducted according to the ethical standards established by the Declaration of Helsinki.

## Results

In 998 cases (89.2%) the police department was responsible for the victim’s referral. The remaining 120 cases (10.8%) sought care spontaneously or were referred by primary care units in the metropolitan region of São Paulo. The average age of the 1,118 victims was 21.0 years ± 5.8 years. The average age of the adolescent group was 15.6 years, with 170 cases (31.1%) aged between 10 and 14 years and 376 cases (68.9%) aged between 15 and 19 years. The average age for the adult group was 26.5 years, with ages ranging from 20 to 62 years. Women of reproductive age, between 20 and 45 years, accounted for 552 cases (96.5%).

In adolescents the frequency of crimes without vaginal, oral or anal penetration was five times higher (Table 1). Rape of a vulnerable person was almost four times more common among adolescents. Being aged under 14 years was predominantly responsible for status as a vulnerable person in adolescents, whereas in the adult group mental illness was the most common factor.

**Table 1:** Comparison of characteristics of sexual violence against adolescent and adult women

	**Adolescents**		**Adults**		**Total**			
**Type of act performed**	**n**	**%**	**n**	**%**	**n**	**%**	***p Value***	**CI 95%**
Vaginal	323	59.1	355	62.0	678	60.6	0.320	0.89 (0.69-1.13)
Vaginal and anal	83	15.2	69	12.0	152	13.6	0.125	1.31 (0.91-1.87)
Vaginal and oral	51	9.3	50	8.7	101	9.0	0.726	1.08 (0.70-1.65)
Vaginal anal and oral	39	7.1	58	10.1	97	8.7	0.075	0.68 (0.44-1.06)
Anal	24	4.4	27	4.7	51	4.5	0.794	0.93 (0.51-1.69)
Oral	7	1.2	9	1.5	16	1.4	0.681	0.81 (0.27-2.40)
No penetration	19	[3.5]	4	[0.7]	23	2.0	[0.001]	5.12 (1.63-17.88)
Total	546	100	572	100	1,118	100		
**Intimidation**	**n**	**%**	**n**	**%**	**n**	**%**	***p Value***	**CI 95%**
Serious threat	345	63.1	388	67.8	733	65.5	0.102	0.81 (0.63-1.05)
Physical violence	67	12.2	82	14.3	149	13.3	0.309	0.84 (0.58-1.20)
Threats and violence	97	17.7	92	16.0	189	16.9	0.453	1.13 (0.81-1.56)
Condition of vulnerability	37	[6.7]	10	[1.7]	47	4.2	[< 0.001]	4.09 (1.93-8.86)
Total	546	100	572	100	1,118	100		
**Condition of vulnerability**	**n**	**%**	**n**	**%**	**n**	**%**	***p Value***	**CI 95%**
Age < 14 years	22	59.4	-	-	22	46.8	-	-
Mental disease	13	[35.1]	7	[70.0]	20	42.5	[0.047]	0.23 (0.04-1.26)
Other condition	2	[5.4]	3	[30.0]	5	10.6	[0.025]	0.13 (0.01-1.26)
Total	37	100	10	100	47	100		

CI 95%: confidence interval of 95%.

(-) not applicable.

There were group differences in perpetrator characteristics, uncles and stepfathers were the perpetrator more frequently in crimes against adolescents; partners or former intimate partners were more frequently the perpetrator in the adult group (Table 2). In both groups lone perpetrators were more common, but a perpetrator known or related to the victim was two and ten times more common in adolescents, respectively.

**Table 2:** Characteristics of the perpetrator of the sexual violence against adolescent and adult women

	**Adolescents**		**Adults**		**Total**			
**Perpetrator’s identification**	**n**	**%**	**n**	**%**	**n**	**%**	***p Value***	**CI 95%**
Unknown	395	[72.3]	504	[88.1]	899	80.4	[< 0.001]	0.35 (0.25-0.49)
Known	151	27.6	68	11.9	219	19.6		
Total	546	100	572	100	1118	100		
**Relationship with the victim**	**n**	**%**	**n**	**%**	**n**	**%**	***p Value***	**CI 95%**
Related	63	[41.7]	9	[13.2]	72	32.9	[< 0.001]	4.69 (2.06-10.99)
Non related	88	58.3	59	86.8	147	67.1		
Total	151	100	68	100	219	100		
**Perpetrator’s typification**	**n**	**%**	**n**	**%**	**n**	**%**	***p Value***	**CI 95%**
Biological father	21	13.9	6	8.9	27	12.3	0.289	1.67 (0.60-4.89)
Stepfather	16	[10.6]	0	[0]	16	7.3	[0.005]	1.50 (1.36-1.66)
Maternal/paternal uncle	14	[9.4]	1	[1.4]	15	6.8	[0.034]	6.85 (0.91-142.43)
Paternal/maternal grandfather	0	0	1	1.4	1	0.4	0.135	0.00 (0.00-7.83)
Brother	7	4.6	0	0	7	3.2	0.071	1.47 (1.34-1.61)
Cousin	5	3.4	1	1.4	6	2.7	0.440	2.29 (0.25-52.93)
Resident in the community	42	27.8	19	27.9	61	27.8	0.984	0.99 (0.50-1.98)
Former intimate partner	9	[5.9]	10	[14.8]	19	8.6	[0.033]	0.37 (0.13-1.04)
Current intimate partner	4	[2.6]	7	[10.4]	11	5.0	[0.016]	0.24 (0.06-0.95)
Known from work	8	5.3	5	7.3	13	5.9	0.551	0.70 (0.20-2.60)
Other acquaintance	25	16.5	18	26.5	43	19.7	0.087	0.55 (0.26-1.16)
Total	151	100	68	100	219	1,000		
**Number of perpetrators**	**n**	**%**	**n**	**%**	**n**	**%**	***p Value***	**CI 95%**
Unique	500	91.6	527	92.1	1,027	91.8	0.733	0.93 (0.59-1.46)
Multiple	46	8.4	45	7.9	91	8.2		
Total	546	100	572	100	1,118	100		

São Paulo. 2012

CI 95%: confidence interval of 95%.

The approach to the victim occurred in public places in 80.5% of cases. Being approached whilst commuting to work or school was more common for adult women, whereas an approach in the residence of the perpetrator was three times more frequent in adolescents (Table 3).

**Table 3:** Activity or situation of the adolescent and adult women at the moment of the sexual assault

	**Adolescents**		**Adults**		**Total**			
**Activity or situation**	**n**	**%**	**n**	**%**	**n**	**%**	***p Value***	**CI 95%**
Displacement from school or work *	155	[28.4]	228	[39.9]	383	34.2	[< 0.001]	0.60 (0.46-0.77)
Daily activity	190	34.8	177	30.9	367	32.8	0.170	1.19 (0.92-1.54)
Victim’s residence	83	15.2	76	13.3	159	14.2	0.359	1.17 (0.82-1.66)
Perpetrator’s residence	23	[4.2]	8	[1.4]	31	2.8	[0.004]	3.10 (1.31-7.60)
Leisure activity	82	15.0	69	12.1	151	13.5	0.148	1.29 (0.90-1.84)
Workplace	10	1.8	14	2.4	24	2.1	0.477	0.74 (0.30-1.80)
Unknown	3	0.6	0	0	3	0.3	0.075	2.05 (1.93-2.18)
Total	546	100	572	100	1,118	100		
Place**	**n**	**%**	**n**	**%**	**n**	**%**	***p Value***	**CI 95%**
Public place	427	78.6	474	82.8	901	80.8	0.073	0.76 (0.56-1.04)
Private place	116	21.4	98	17.1	214	19.2		
Total	543	100	572	100	1,115	100		

São Paulo. 2012.

CI 95%: confidence interval of 95%.

(*): displacement from school or work.

(**): excluded three cases of unknown place of the sexual violence due to condition of vulnerability.

## Discussion

Although a woman may be the victim of sexual violence at any age, most studies report a higher frequency of offences against children and teenagers, most of them of reproductive age, as observed in this study [15]. The frequencies of sex crimes including vaginal, anal or oral penetration did not differ between groups (Table 1). The 1,095 adolescents and adult women (97.5%) were similarly subjected to penetrative acts during sex crimes, but sexual crimes involving children were mostly non-penetrative sexual abuse [16].

Crimes involving vaginal penetration occurred in 1,028 cases (91.9%); anal penetration was involved in 51 cases (4.5%), a finding which differs from data reported by São Paulo forensic medical departments in who recorded lower frequencies of vaginal penetration (60%) and more anal penetration (18%) during the same period [17].

Non-penetrative sexual crimes occurred five times more frequently in the adolescent group (Table 1). However, this effect was much smaller than that seen in most studies with children, who were the victim in almost half of these cases [18].

Sexual violence involving vaginal intercourse without condom use affected 928 patients (83.0%) of reproductive age (15-44 years), a similar rate to that found in other studies (Table 1) [19]. Under these circumstances the sexual violence subjects the victim to the risk of unwanted pregnancy unless she had effective contraception protection independent of the perpetrator [13].

Likewise, in 1,079 cases (96.5%) unprotected vaginal or anal penetration exposed adult and adolescent victims to STDs including viral hepatitis and HIV [16]. The risk for these diseases was higher in 97 cases (14.0%), which involved simultaneous vaginal and anal penetration during sexual violence (Table 1).

Although the legal definitions of sexual violence vary according to the laws of the country, most legal codes recognize that use of physical force, intimidation and lack of consent constitutes a crime [20]. Psychological intimidation by threatening death or serious physical harm occurred in 733 cases (65.5%) and with similar frequency in both groups (Table 1), consistent with findings from other studies [15,16,20,21]. The serious threat or extreme fear generated in these situations may be associated with human tonic immobility, leading to complete inability to offer physical resistance to the perpetrator [22]. Threats with guns can inhibit the woman’s resistance, reducing significantly the physical damage she suffers [23]. In addition to the implications for health, physical trauma constitutes important forensic evidence in sex crimes cases [16,24].

Situations of legally recognized vulnerability were almost four times more frequent in adolescents, especially for vulnerability by reason of being aged under 14 years (Table 1). In such cases, sexual contact is characterized as abusive based on the psychosocial immaturity of the adolescent and hence her inability to consent [25]. However, these cases make up only 12.9% of the case involving adolescents aged 10-14 years, indicating that use of physical force and/or the threat of physical force is still the dominant mode of sexual abuse in this age group. Age as a vulnerability factor was less frequent in this study than in studies with children under the same conditions, when it approaches 60% of sexual violence cases [16,23].

Rapes involving women with mental illness were twice as common in the adult group (Table 1). It is estimated that almost fifty percent of women with severe mental illness will suffer sexual violence at least once during their life [26]. It is possible that the limited autonomy of these patients and their association with known or related perpetrators contributes to sexual violence remaining hidden and underreported, which may explain the small number of cases found in this study [27].

Other conditions of vulnerability recognized by Brazilian law were found in a smaller percentage of cases (Table 1). These include the use of alcohol, severe and debilitating diseases, and physical disabilities [25]. The use of drugs that act on the central nervous system is also covered by this law. Usually such drugs are used without the victim’s knowledge in order to reduce consciousness and decrease resistance, thereby facilitating sexual crime [25,28]. Benzodiazepines are the drug most commonly used for this purpose, identified in 82% of cases of sexual violence involving drugs.

A majority of perpetrators were unknown to the victim in both the adult group (88.1%) and the adolescent group (72.3%). It is possible that some of the victims have concealed the identity of the perpetrator fearing retaliation for reporting the violence. This is the more plausible when the fact that 82.4% of the cases involved some relevant form of threat is taken into account (Table 2).

In contrast to findings from this study, perpetrators known to the victim are commonly reported in sexual offences against adolescent and young adult women [29-31]. In some studies, unknown perpetrators are involved in up to 70% of cases of sexual violence, similar to our data [19,32]. It is considered that underreporting of sexual violence may be more common when the perpetrator is related or known to the victim. The sample and study design may be critical to identifying situations in which sexual violence is committed by perpetrators known to the victim [32].

Although unknown perpetrators have been reported by most teenagers (Table 2), there were often twice the number of perpetrators identified in this group (27.6%). Furthermore in the adolescent group perpetrators related to the victim were reported almost eight times more frequently than in the adult group. These features show a similarity to the characteristics of childhood sexual abuse, which is usually perpetrated by adults the child knows and trusts [18]. In Mexico City, 86.7% of known perpetrators were relatives or friends of the family [30]. This privileged position generates different barriers to reporting sexual crime, which can be a decisive factor in its continuation until adolescence or early adulthood [18].

The characteristics of perpetrators who were known to the victim underlines group differences (Table 2). While uncles and stepfathers were more common perpetrators against adolescents (20.0%), partners or former intimate partners were more common perpetrators against adult women (25.2%). Although they have relevant participation among known offenders, there is evidence that underreporting is higher in cases where the perpetrator is known to the victim [14,16].

Cultural factors lead almost 30% of Brazilian women to believe they have an obligation to have sex when partners seek it, even if they do not want it [33]. In rural communities in Nepal, 42% of young married women admitted that their husbands used physical force to have sex with them [5]. A survey of almost 1,400 young men from 70 communities in South Africa showed that 16% practiced sexual acts against the will of their partner and that 8% admitted to using some form of violence during intercourse [34].

In 8.1% of cases more than one perpetrator participated in the violence (Table 2). In these cases the number of unknown offenders varied from two to six. Multiple offender cases are reported less frequently in other studies, typically accounting for about 20% of cases of sexual violence [15]. Psychological sequelae may be more severe when the crime involves multiple perpetrators. It is also believed that the higher the number of perpetrators the greater the risk of transmission of STDs including HIV [16].

The place where victims were approached, or their activity when approached, appears to be closely related to the characteristics of the perpetrator (Table 3). Public spaces were the site of the approach with similar frequency in both groups, consistent with the high frequency of perpetrators unknown to the victim. The number of women approached on their way to work (34.2%) was particularly striking.

It is even more complex and difficult to report sexual crimes committed by intimate partners, where the approach usually occurs domestically, this explains the very small number of cases in this study [36]. The lower recognition by women of domestic sexual violence and the risks they face from an intimate partner may contribute to underreporting of this type of crime. This also makes it difficult to provide appropriate care services for victims of these sexual offences [1,33-37].

Crimes committed in the perpetrator’s residence were three times more frequent in the adolescent group, when compared to other studies’ findings [16,17,19]. An approach in private space was less frequent in the adult group, suggesting greater vulnerability of adolescents to offenders belonging or related to their nuclear family, giving these cases a similarity to cases of childhood sexual abuse.

The vulnerability of adolescents to sexual crimes suggests that they require the same legal protection measures that are applied to children, the high rate of intrafamilial sexual abuse provides supporting evidence for this need. However, this study found a significant proportion of adolescents face sexual violence in situations similar to those experienced by adult women, something that has been less frequently reported in the literature. This seems to be characteristic of urban situations, consistent with the prevalence of perpetrators unkonwn the victim, who approached or threatened the victim in public spaces.

Our findings suggest that public health services trained in the treatment of sexual crimes can be identified by the victims as local host, support and care. At the same time, that sex crimes committed by known or related perpetrators in private spaces was the lowest frequency category in the adolescent group pointing to the limited ability of the health service to act in cases of intrafamilial sexual abuse.

The most important limitation of our study is that the data were collected retrospectively from existing patient files, a method which carries the usual limitations of recorded patient information, including certain gaps and possible errors.

## Conclusion

Sexual crimes showed similar characteristics in both age groups studied. A significant portion of the cases was associated to acts of unprotected vaginal penetration, implying risk of unwanted pregnancy and sexually transmitted diseases. Unknown perpetrators used mainly the threat of death to intimidate the victims, usually addressed in public spaces during daily activities. Among the situations of vulnerability established by legislation, mental illness was more common among adolescents, whereas adult women prevailed in other forms of prevention of resistance or consent.

## Competing interests

The authors declare that they have no competing interests.

## Authors’ contributions

MTB, JD, MAV, FA, VEV, ACP, JMV, DP and LCA participated in the acquisition of data and revision of the manuscript. MTB, JD, MAV, ACP and DP conceived of the study, determined the design. VEV, LCA, FA, ACP, MAv, MTB, and JD performed the statistical analysis, interpreted the data and drafted the manuscript. All authors read and gave final approval for the version submitted for publication.

## Pre-publication history

The pre-publication history for this paper can be accessed here:

http://www.biomedcentral.com/1472-6874/14/15/prepub

## References

[B1] Krug EG Dahlberg LL Mercy JA Zwi AB Lozano R World report on violence and health 2002 Geneva: World Health Organization (WHO)

[B2] Drezett J Kurobe FC Nobumoto CT Pedroso D Blake MT Valenti VE Hydatidiform mole resulting from sexual violence Int Arch Med 2012 5 8 10.1186/1755-7682-5-8 22353179 PMC3296577

[B3] Nations U Report of the Fourth World Conference on Women, Beijing 1995 New York: United Nations

[B4] Drezett J Pedroso D Vertamatti MA Macedo-Júnior H Blake MT Gebrim LH Pregnancy resulting from sexual abuse: reasons alleged by Brazilian women for carrying out the abortion – pregnancy and violence HealthMED 2012 6 819 825

[B5] Lamichhane P Puri M Tamang J Dulal B Women’s status and violence against young married women in rural Nepal BMC Womens Health 2011 11 19 10.1186/1472-6874-11-19 21612603 PMC3121674

[B6] Beebe DK Sexual assault: the physician’s role in prevention and treatment J Miss State Assoc 1998 39 366 369 9796180

[B7] Sapp MV Vandeven AM Update on childhood sexual abuse Curr Opin Pediatr 2005 17 258 264 10.1097/01.mop.0000158731.64293.c7 15800423

[B8] Vertamatti MAF de Abreu LC Drezett J Valenti VE Barbosa CP Time lapsed between sexual aggression and arrival at the brazilian health service J Hum Growth Dev 2013 23 46 51

[B9] Mulugeta E Kassaye M Berhane Y Prevalence and outcomes of sexual violence among high school students Ethiop Med J 1998 36 167 174 10214457

[B10] Singh HS Yiing WW Nurani HN Prevalence of childhood sexual abuse among Malaysian paramedical students Child Abuse Negl 1996 20 487 492 10.1016/0145-2134(96)00030-0 8800523

[B11] Coker AL Richter DL Violence against women in Sierra Leone: frequency and correlates of intimate partner violence and forced sexual intercourse Afr J Reprod Health 1998 21 61 72 10214430

[B12] Shamu S Abrahams N Temmerman M Musekiwa A Zarowsky C A systematic review of african studies on intimate partner violence against pregnant women: prevalence and risk factors PLoS One 2011 6 e17591 10.1371/journal.pone.0017591 21408120 PMC3050907

[B13] Ministério da Saúde (Brasil) Secretaria de Atenção à Saúde. Departamento de Ações Programáticas Estratégicas Área Técnica de Saúde da Mulher. Prevenção e tratamento dos agravos resultantes da violência sexual contra mulheres e adolescentes: norma técnica 2011 3 Brasília: Editora MS

[B14] Mattos IA Lima IMS Motherhood and intrafamilial child sexual abuse: to guarantee a protective embrace J Hum Growth Dev 2012 22 373 377

[B15] Avegno J Mills TJ Mills LD Sexual assault victims in the emergency department: analysis by demographic and event characteristics J Emerg Med 2009 37 328 334 10.1016/j.jemermed.2007.10.025 18394848

[B16] Drezett J Caballero M Juliano I Prieto ET Marques JA Fernandes CE Study of mechanisms and factors related to sexual abuse in female children and adolescents J Pediatr 2001 77 431 439 10.2223/jped.284 14647847

[B17] Cohen C Matsuda NE Sex crimes and forensic sexology: analytic study Rev Paulista Med 1991 109 157 164 1775883

[B18] Daneri RA Urueña-Tincani EM Góngora AM Boscarino GL Sexual abuse in children Med Infant 2008 15 312 319

[B19] Boykins AD Mynatt S Assault history and follow-up contact of women survivors of recent sexual assault Issues Ment Health Nurs 2007 28 867 881 10.1080/01612840701493394 17729171

[B20] Heinrich LB Care of the female rape victim Nurse Pratic 1987 12 9 12 3320827

[B21] Aguilar A Salcedo M Characteristics of sexual violence in adolescents from 10 to 19 years of age, Cali 2001-2003 Colomb Med 2008 39 356 363

[B22] Abrams MP Carleton RN Taylor S Asmundson GJ Human tonic immobility: measurement and correlates Depress Anxiety 2009 26 550 556 19170102 10.1002/da.20462

[B23] Reis JN Martin CCS Ferriani MGC Female victims of sexual abuse: coercive methods and non-genital injuries Cad Saude Publica 2004 20 465 473 10.1590/S0102-311X2004000200014 15073626

[B24] Biggs M Stermac LE Divinsky M Genital injuries following sexual assault of women with and without prior sexual intercourse experience CMAJ 1998 159 33 37 9679484 PMC1229478

[B25] Ministério da Saúde (Brasil) Juridical aspects of the attention to victims of sexual violence: questions and answers for health professionals 2010 2 Brasília: Editora do Ministério da Saúde

[B26] Zanarini MC Frankenburg FR Reich DB Marino MF Haynes MC Gunderson JG Violence in the lives of adult borderline patients J Nerv Ment Dis 1999 187 65 71 10.1097/00005053-199902000-00001 10067945

[B27] Lumley VA Miltenberger RG Long ES Rapp JT Roberts JA Evaluation of a sexual assault abuse prevention program for adults with mental retardation J Appl Behav Anal 1998 31 91 101 10.1901/jaba.1998.31-91 9532753 PMC1284101

[B28] Du Mont J Macdonald S Rotbard N Asllani E Bainbridge D Cohen MM Factors associated with suspected drug-facilitated sexual assault CMAJ 2009 180 513 519 10.1503/cmaj.080570 19255075 PMC2645446

[B29] Djezzar S Questel F Burin E Dally S Chemical submission: results of 4-year French inquiry Int J Legal Med 2009 123 213 219 10.1007/s00414-008-0291-x 18925406

[B30] Garza-Aguilar J Díaz-Michel E Elements for the study of rape Salud Publica Mex 1997 39 539 545 9477736

[B31] Rickert VI Wiemann CM Date rape among adolescents and young adults J Pediatr Adolesc Gynecol 1998 11 167 175 10.1016/S1083-3188(98)70137-8 9806126

[B32] Muganyizi PS Kilewo C Moshiro C Rape against women: the magnitude, perpetrators and patterns of disclosure of events in Dar es Salaam, Tanzania Afr J Reprod Health 2004 8 137 146 17348331

[B33] Faúndes A Hardy E Osis MJ Duarte G Risk of gynecologic complaints and sexual dysfunctions according to history of sexual violence Rev Bras Ginecol Obstet 2000 22 153 157

[B34] Jewkes R Dunkle K Koss MP Levin JB Nduna M Jama N Rape perpetration by young, rural South African men: prevalence, patterns and risk factors Soc Sci Med 2006 63 2949 2961 10.1016/j.socscimed.2006.07.027 16962222

[B35] Ramos CRA Medicci VPG Puccia MIR Sexually abused women: social and demographic profile and health care procedure analysis at a referral center J Health Sci Inst 2009 7 22 27

[B36] Atrash HK Carpentier R The evolving role of public health in the delivery of health care J Hum Growth Dev 2012 22 396 399

[B37] Souza e Silva R Prevalence and characteristics of women with abortion among women with history of pregnancies J Hum Growth Dev 2012 22 27 33

